# A magnetic solid phase chemiluminescent immunoassay for quantification of Cystatin C in human serum

**DOI:** 10.1186/s12896-023-00813-6

**Published:** 2023-10-11

**Authors:** Jian He, Ping Liang, Tingting Wang, Shuang Han

**Affiliations:** 1 Department of Clinical Laboratory, Affiliated Children’s Hospital of Jiangnan University, Wuxi, China; 2 Wuxi School of Medicine, Jiangnan University, Wuxi, China; 3 Department of Clinical Laboratory, Wuxi People’s Hospital Affiliated to Nanjing Medical University, Wuxi, China; 4 Department of Pathology, Affiliated Hospital of Jiangnan University, Wuxi, Jiangsu 214062 China

**Keywords:** Cystatin C, Chemiluminescent immunoassay, Quantification detection, Kit development

## Abstract

A chemiluminescent immunoassay for human serum Cystatin C (Cys C) was established using a direct-antibody sandwich model. The immunoassay kit uses magnetic separation technology, using magnetic particles as the reaction solid phase, alkaline phosphatase as the marker enzyme, and a new chemiluminescent substrate APLS as the substrate. It has the characteristics of high sensitivity and short reaction time. This product uses high-affinity antibodies, resulting in a high specificity. The established method showed good accuracy, uniformity, and stability. The limit of detection was 2.39 ng/mL. The intra-assay coefficient of variation (CV) was 3.36%–6.00%, the interassay CV was 4.12%–5.35%, and the recovery rate was 99.07%. The correlation coefficient (*r*) of Cys-C kit was 0.999388 ≥ 0.9900. The accuracy of the developed method was tested by automatic chemiluminescence instrument (*P* > 0.05). The lowest titer was 0.92500, and the highest was 1.10000. The developed method showed a good correlation with the product from Roche by comparing these two kits in 240 clinical samples from China. In total, 1392 clinical patient from China samples were measured using the reagent kit developed in this study.

## Introduction

Clinical evaluation of renal disease progression and severity is generally based on renal function, which is generally reflected by glomerular filtration rate (GFR) [1–3]. GFR is a rate, that represents the flow of plasma from the glomerulus into Bowman’s space over a specified period and is the chief measure of kidney function [2]. GFR is the amount of plasma that passes through the glomeruli over a given period of time (defined as the amount of plasma that the kidneys clear from the body per unit of time, usually in (mL/min) [4, 5]. It can not be measured directly and must be reflected by the kidney clearance of a substance [1]. According to the source of GFR markers, it can be divided into exogenous and endogenous. The determination method of exogenous marker kidney clearance rate is regarded as the "gold standard" of GFR evaluation [1, 6]. However, there are many shortcomings, for example, the detection methods of colloidal gold method and immunochromatography were complex and the detection result is insensitive, inaccurate, imprecise [7]. Endogenous markers are the most commonly used indicators in evaluating glomerular filtration function [7, 8]. Fortunately, Serum Cys C basically met the requirements of ideal endogenous GFR markers, and was thus used as a new sensitive indicator to evaluate GFR [9–11].

Nowadays, the well-established endogenous biomarker for GFR includes Cys-C, serum creatinine (SCr), creatinine clearance (CCr) and β2-microglobulin (β2-MG). SCr levels were reported to be influenced by the muscle mass, age and sex [12]. More importantly, 60–70% of kidney functional units were already damaged when SCr levels were aberrantly upregulated which suggested that SCr levels were lack of sensitivity [13]. During clinical diagnosis, urine collection and storage conditions could cause large differences in CCr levels which indicated that CCr levels were not stable enough [14]. As for β2-MG, its levels were affected by multiple factors, like inflammation, haematopoietic cell changes, malignancy, and immunosuppressive agents [14, 15]. These descriptions were added in the introduction and highlighted in red. Here, Cys C is one of the cysteine protease inhibitor proteins, also known as γ-microprotein and γ-postglobulin [16–18]. The gene encoding Cys C belongs to the steward gene, which can be continuously transcribed and expressed in all nuclear cells at a constant rate without tissue specificity [16]. Notably, unlike above mentioned factors, Cys C can be produced at a constant rate in the body and coexist in various body fluids, especially in cerebrospinal fluid and seminal plasma, with the lowest content in urine, which is not affected by age, gender, weight, inflammation and other factors [16, 19, 20]. It is a low molecular weight, basic, non-glycosylated protein encoded by the CST3 gene with a molecular weight of 13.3 kDa, consisting of 122 amino acid residues [16]. It also carries a positive charge under physiological conditions, can be freely filtered out of the glomeruli, is completely reabsorbed by the renal tubular epithelial cells and degraded intracellularly, and does not return to the blood [16]. Notably, it is an endogenous marker reflecting changes in GFR and reabsorbed in the proximal tubule, but is completely metabolized and decomposed after reabsorption and does not return to the blood [9, 21]. Compared with creatinine, its blood concentration is only determined by glomerular filtration, and is not influenced by any external factors, such as gender, age or diet [21, 22]. Therefore, it is an ideal homologous marker to reflect changes in GFR [9, 23]. Currently most of the methodologies used for the clinical detection of Cys-C are immunochromatographic and colloidal gold methods. The fact that these two methods are mainly based on solid-phase reactions leads to a lack of sensitivity, accuracy and precision in clinical applications [24, 25]. In contrast to these two methods, chemiluminescence is based on liquid-phase reaction which makes it can give sufficient contact area and chance between substrate and reactants and therefore has obvious advantages in linear range, sensitivity, precision, accuracy and good detection stability, and the detection is fast and convenient with fully automated instruments [26]. In this study, a sandwich chemiluminescence assay was established to test the Cys C in the human serum.

## Materials and methods

In brief, this section mainly demonstrated the composition of the kit (Reagents and Materials section), preparation process of the relevant reagents (Reagent Component Preparation section) and the operating Procedure of the kit (Method Procedure section).

### Reagents and materials

Cys-C antibody conjugations for anti-reagent production, including fluorescein isothiocyanate (FITC)-labeled Cys-C antibody conjugations (Anti-Cys-C FITC, 0.8 μg/mL) and alkaline phosphatase (AP)-labeled Cys-C antibody conjugations (Anti-Cys-C AP, 0.5 μg/mL), magnetic particle coupling of anti-fluorescein isothiocyanate antibody (magnetic particle-anti-FITC antibody) and Cys-C recombinant antigen were purchased from Beijing Zecheng Biotechnology (Beijing, China); BSA is purchased from ROCHE (Germany); newborn calf serum is purchased from GIBCO (New York, USA); chemiluminescent substrate solution for automatic immunoassay, cleaning solution for chemiluminescent system, pipeline cleaning solution are purchased from Zecheng Biotechnology (Beijing, China); Magnetic particle reagent are purchased from BIOMAG Biotechnology (Wuxi, China); an automatic chemiluminescence analyzer is supplied by Zecheng Biotechnology from China.

### Reagent component preparation

#### Requirements for the sample

This kit uses serum as the test specimen, and it is recommended to collect 5.0 ml of venous blood into a glass tube and either stand at room temperature or centrifuge to separate the serum portion. Avoid using serum samples with severe hemolysis, hyperlipidemia and high alkaline phosphatase activity. The sample size should not be less than 200 *μ*L, and the serum sample should be diluted as 1: 100 with sample diluent.

#### Characterisation of Cys-C antibody conjugate

Anti-reagent A: The Anti-Cys-C-FITC antibody conjugate labeled by fluorescein isothiocyanate (FITC) is dialysed into BSA buffer (pH = 8.0) in a certain concentration ratio. Anti-reagent B: alkaline phosphatase (AP) labeled Cys-C antibody conjugate (Anti-Cys-C-AP) is dialysed into BSA buffer (pH = 8.0) in a certain concentration ratio. The absorbance of the antibody was measured at 280 nm by ultraviolet spectrophotometer, and the concentration and recovery rate were calculated. The concentrations of Anti-Cys-C-FITC and Anti-Cys-C-AP were 1.45 mg/mL and 2.3 mg/mL, and the recoveries were 102.07% and 103.04%, respectively. The function of the Cys-C antibody conjugate was detected by the confirmed sandwich method. The concentration of Anti-Cys-C-AP was 0.5 μg/mL, when the concentration of Anti-Cys-C-FITC was 0.8 μg/mL, the curve gradient was good. The results showed that the antibody pair was suitable for this detection system with strong specificity and high sensitivity (Table 1).

**Table 1:** Optimisation of antibody concentration

Anti-Cys-C-FITC (*μ*g/mL)		0.5			0.8				2.0
Anti-Cys-C-AP (*μ*g/mL)	0.5	0.8	2.0	0.5	0.8	2.0	0.5	0.8	2.0
Standard 1 (0.0 μg/mL)	3228	3206	3185	3674	4143	6254	3260	4155	3911
Standard 2 (0.2 μg/mL)	35,958	36,370	38,082	155,108	159,117	172,135	68,499	71,469	76,290
Standard 3 (6.0 μg/mL)	395,290	428,691	417,072	2,396,293	2,606,007	2,649,074	813,664	1,043,755	1,082,789

#### Characterisation of Cys-C antigen

The Cys-C antigen concentration was measured by the immunoassay kit, and the EKONZYME magnetic separation enzyme-linked quantitative immunoassay kit was used for detection. The recovery rate of the selected Cys-C antigen was 99.07%.

#### Characterisation of Cys-C calibrators and quality control products

Accurately weigh a certain amount of Cys-C dissolved in BSA buffer to produce Cys-C calibrator (pH = 7.5) /quality (pH = 7.0) control product concentrate for use; buffers containing bovine serum were added with different amounts of Cys-C calibrators/quality control product concentrates to produce a calibrator/quality control product of the specified concentration. The calibrators/quality control products were assigned using the working reference of national Standard product traceability (WRS).

#### General reagent production process

The luminescence gradients of each point of the calibrator at different concentrations were compared. Finally, the working concentration of magnetic particle reagent was determined to be 3.0 mg/mL, the content of BSA and Tween 20 added to the buffer of magnetic particle reagent were determined to be 0.5% and 0.25%.

#### Optimisation of reaction conditions

This kit uses the direct sandwich principle, where fluorescein isothiocyanate (FITC) labeled Cys-C antibody is combined with alkaline phosphatase (AP) labeled Cys-C paired antibody to form a "sandwich" complex with Cys-C in the sample, calibrator or quality control product. The antigen–antibody immune complex was bound to the magnetic particle with anti-FITC antibody as the solid phase of the immune response through the specific binding of anti-FITC antibody and FITC. The luminescence intensity of the reaction was measured by chemiluminescence enzyme-linked immunoassay and chemiluminescence apparatus. Within the detection range, the luminescence intensity was proportional to the content of Cys-C in the sample, and the concentration of Cys-C in the sample could be calculated using the modified four-parameter Logistic equation fitting. In this study, sample size, reaction temperature, reaction system and other conditions were optimized.

### Method procedure

Add 20 *μ*L of calibration, test samples and quality control products into the reaction cup, then add 30 *μ*L of anti-reagent A (anti-Cys-C-FITC) and 30 *μ*L of anti-reagent B (anti-Cys-C-AP), mix well and react at 37℃ temperature bath for 5 min, add 30 *μ*L of magnetic particle reagent, mix and react at 37℃ temperature bath for 5 min, add 300 *μ*L of cleaning solution for chemiluminescence system, mix well, Repeat this step 3 times for magnetic separation and cleaning of the reactants. Finally, add chemiluminescent substrate solution for automatic immunoassay for luminescence value detection and concentration values are calculated if the calibrator curve is available.

## Performance test method

### Limitation of detection

The zero calibrator was repeated for no less than 10 times, and the mean $\left(\overline{X }\right)$ and standard deviation (SD) of the RLUs were calculated. The reaction amount $\boldsymbol{ }\left({\overline{X} }_{+2SD}\right)$ was substituted into the dose–response curve to calculate the corresponding concentration value.

### Precision and recovery

To test the precise and optimize the detection conditions, two standard samples of different Cys-C concentrations were tested and duplicated separately in one experiment and repeated after 20 days, and the intra-assay and interassay coefficient of variation (CV) were calculated. Concentrated Cys-C solutions were added to three serum samples with different analyte levels, and the recovery rate was calculated.

### Linearity of dose–response curve

The high-value samples close to the upper limit of the linear range are diluted to at least 5 concentrations in a certain proportion, among which the low-value samples must be close to the lower limit of the linear range. According to the instructions of the kit, the sample of each concentration was repeated for 3 times, and the mean value was calculated. The mean value of the result and the dilution ratio were fitted linearly by the least square method, and the linear correlation coefficient r was calculated, and the correlation coefficient r of Cys-C kit was 0.999388 ≥ 0.9900.

### Accuracy

The Cys-C national standard was prepared into concentrations corresponding to the calibrators in the kit using the kit buffer liquid system. Each parallel measurement was performed no less than twice, and the slopes and titer ratios of the two dose–response curves were calculated. The accuracy of the kit was tested by automatic chemiluminescence instrument (*P* > 0.05). The lowest titer was 0.92500, and the highest was 1.10000.

### Accelerated stability

The whole kit including anti-Cys-C-FITC, magnetic particle, anti-Cys-C-AP, and Cys-C standards was incubated at 37 ℃ for 7 days, and the signals of standards and samples at different days were compared.

### Method comparison

The established method was compared with the market CLIA method from Roche. In total, 240 samples were used, ranging from 0.57 to 9.99 μg/mL.

## Results

### Method performance

The 4-parameter logic function method was used to fit the standard curve of signal with Cys-C concentration. The following series concentrations of Cys-C were used as a standard curve (A-F): 0 μg/mL, 0.2 μg/mL, 0.4 μg/mL, 1.0 μg/mL, 3.0 μg/mL, and 6.0 μg/mL. The LoD is ≦ 0.01 μg/ml. Precision was tested, intra-assay CV % is 3.36% – 6.00%, interassay CV % is 4.12% – 5.35%, and the results are shown in Table 2. The recovery rate of Cys-C antigen is 99.07%.

**Table 2:** Precision test results

Samples	Intra-assay CV (*n* = 20)			Interassay CV (*n* = 20)		
	Mean (*μ*g/mL)	SD	CV (%)	Mean (*μ*g/mL)	SD	CV (%)
1	0.42	0.02	6.00	0.42	0.02	5.35
2	3.01	0.10	3.36	3.00	0.15	4.12

### Method optimization

#### Optimization of sample volume

**Fig. 1:**
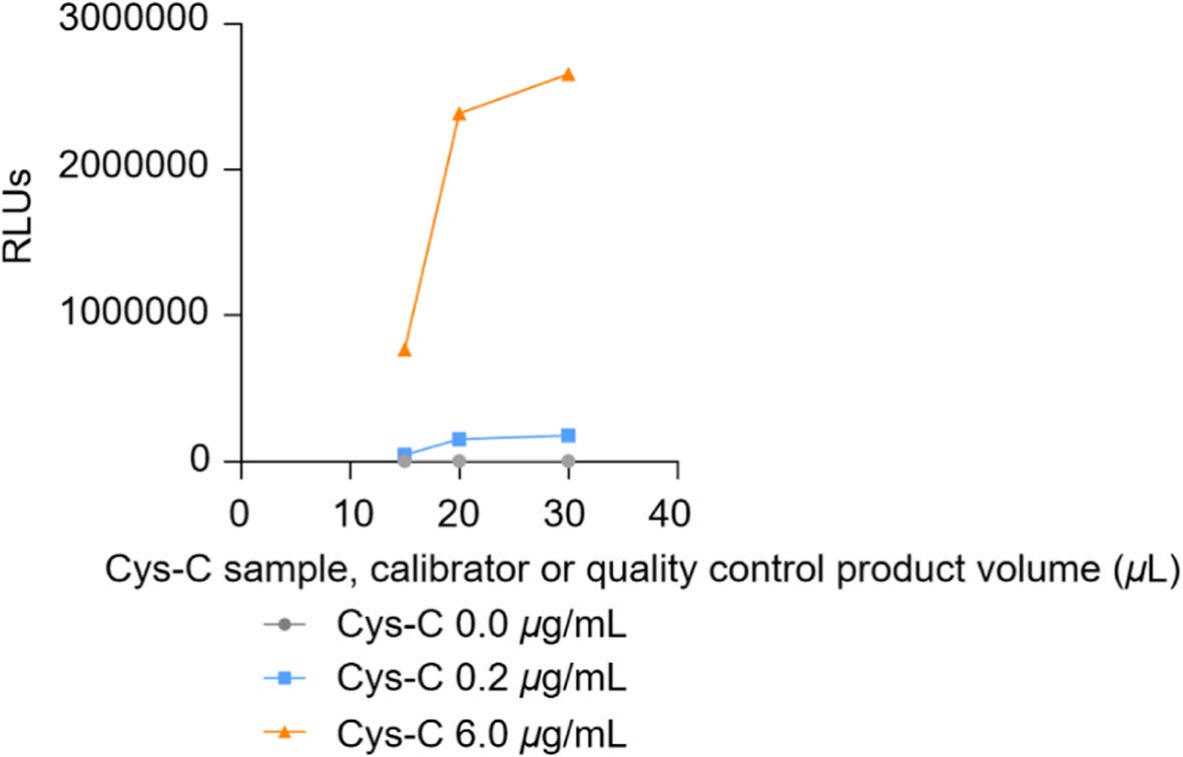
Optimization of the Cys-C sample volume

Cys-C samples were added at 15 *μ*L, 20 *μ*L and 30 *μ*L to test calibration curves respectively. Under other conditions being the same, the curve linearity and luminescence intensity changes under different reagent dosages were compared, and the most suitable dosages were selected, and the results are shown in Fig. 1. According to the experimental results, 20 *μ*L was selected as the addition quantity of samples, calibrators or quality control products of Cys-C.

#### Optimization of adding amount of Cys-C anti-reagent

Three dosages of Cys-C were selected, including 15 *μ*L, 30 *μ*L and 60 *μ*L, and the calibration curves were tested respectively. Under other conditions being the same, the curve linearity and luminescence intensity changes under different reagent dosages were compared, and the most suitable dosages were selected, and the results are shown in Table 3. According to the experimental results, it was determined that the selection of anti-reagent dosage of Cys-C was 30 *μ*L + 30 *μ*L.

**Table 3:** Optimisation of the Cys-C anti-reagent volume

Anti-reagent volume	15 *μ*L + 15 *μ*L	30 *μ*L + 30 *μ*L	60 *μ*L + 60 *μ*L
Standard 1 (0.0 μg/mL)	3547	3574	3586
Standard 2 (0.2 μg/mL)	46,322	151,108	178,878
Standard 3 (6.0 μg/mL)	750,504	2,326,293	2,658,985

#### Optimization of incubation time

**Fig. 2:**
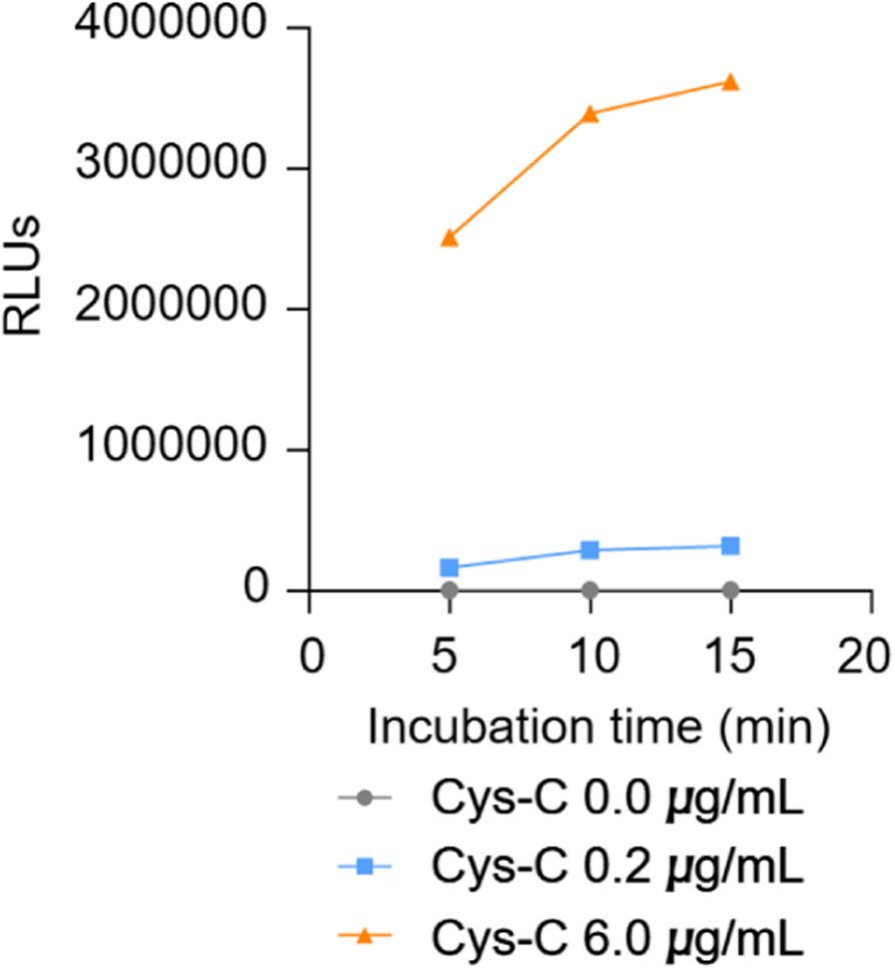
Optimization of incubation time

The reaction time of the reagent and the sample was selected as 5 min, 10 min and 15 min, respectively. The luminescence intensity of the points A, B and F of the calibrator, the opening gradient and the ease of operation were used as the evaluation criteria to select the reaction time. According to the results shown as Fig. 2, when the reaction time reached 5 min, the increase of luminescence value was not significantly affected by the extension of time, and the linearity of calibration curve was not significantly changed. Therefore, the reaction time of anti-reagent detection by Cys-C was 5 min.

### Method comparison

**Fig. 3:**
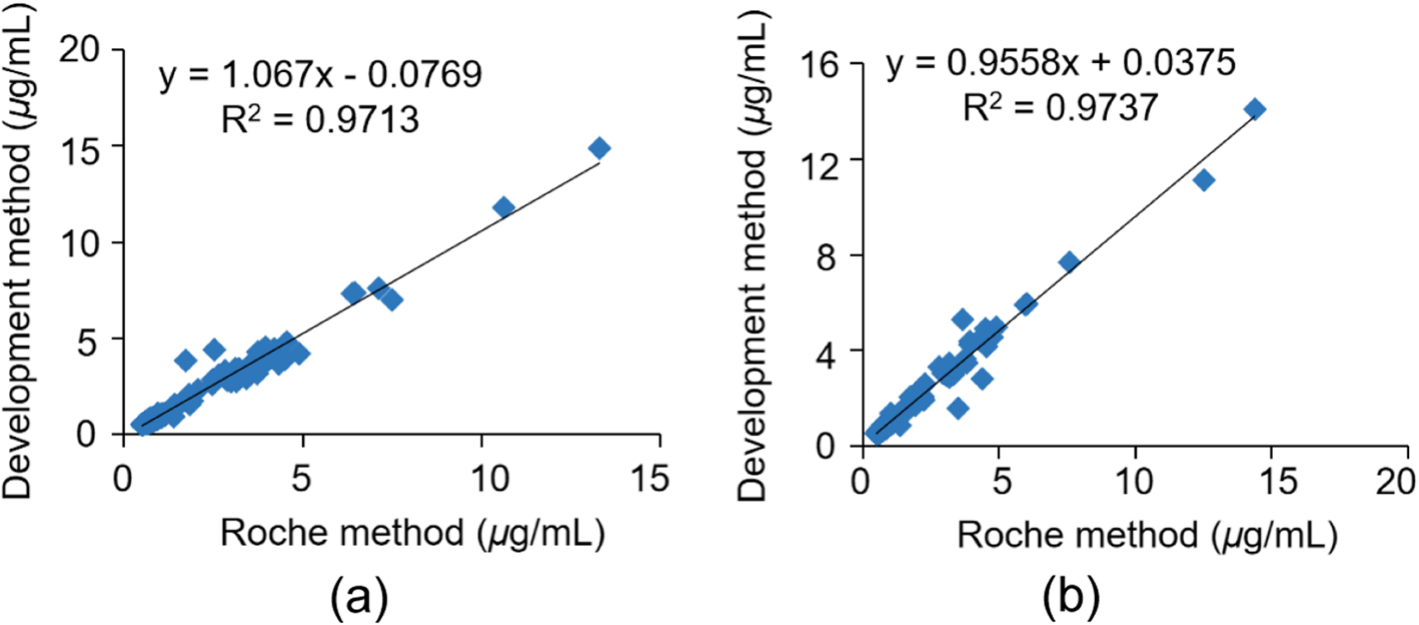
Comparison of the developed method and Roche method. Samples in a were collected from Jilin Neuropsychiatric Hospital and Samples in b were collected from Liaoning Mental Health Center

In this study, 240 human serum samples from two different hospitals were measured using both the developed method and the Roche method. The test results were regressed using the least-squares regression equation, and the correlation coefficient was computed. Data are shown in Fig. 3. The test results show good agreement between the developed and compared methods, and the difference in test and mean values indicated a slight bias between these methods.

### Clinical study

**Fig. 4:**
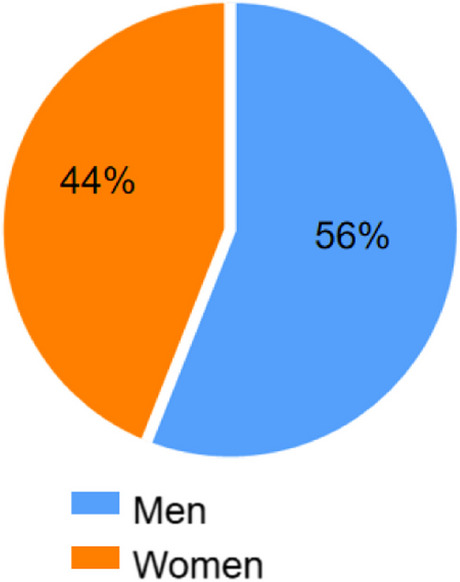
Sex distribution of clinical samples

**Fig. 5:**
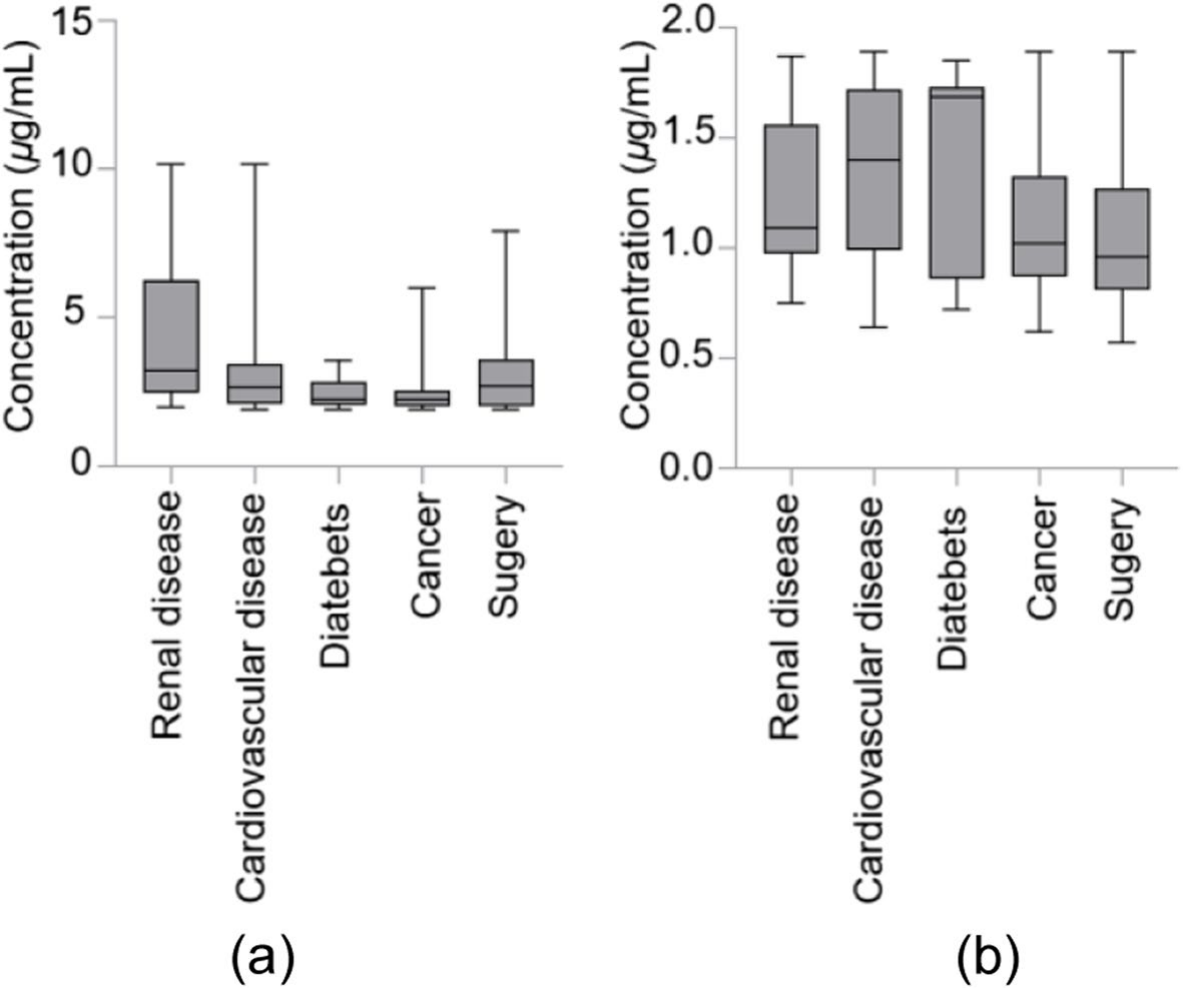
Concentration of Cys-C of different patients. Concentration of Cys-C ≥ 1.90 μg/mL (**a**) and Concentration of Cys-C < 1.90 μg/mL (**b**)

One thousand three hundred ninety-two clinical patient samples were measured with developed reagent kit in this study. 5 types of disease were involved (Table 4). Among patients have high-level Cys-C (≥ 1.9 μg/ml), nearly 20% are related to renal diseases, about 25% patients have cancers, and almost 27% got surgery. The sex ratio of patient samples is shown in Fig. 4. The range of Cys-C concentrations in different patients is shown in Fig. 5.

**Table 4:** Distribution of clinical samples

Conc. of Cys-C (μg/mL)	Cardiovascular disease	Cancer	Renal disease	Diabetes	Surgery
0.5 ~ 1.9	83	525	28	8	472
≥ 1.9	72	68	54	12	12
Total	155	593	70	20	542

### Scheme of the applied procedure

**Fig. 6:**
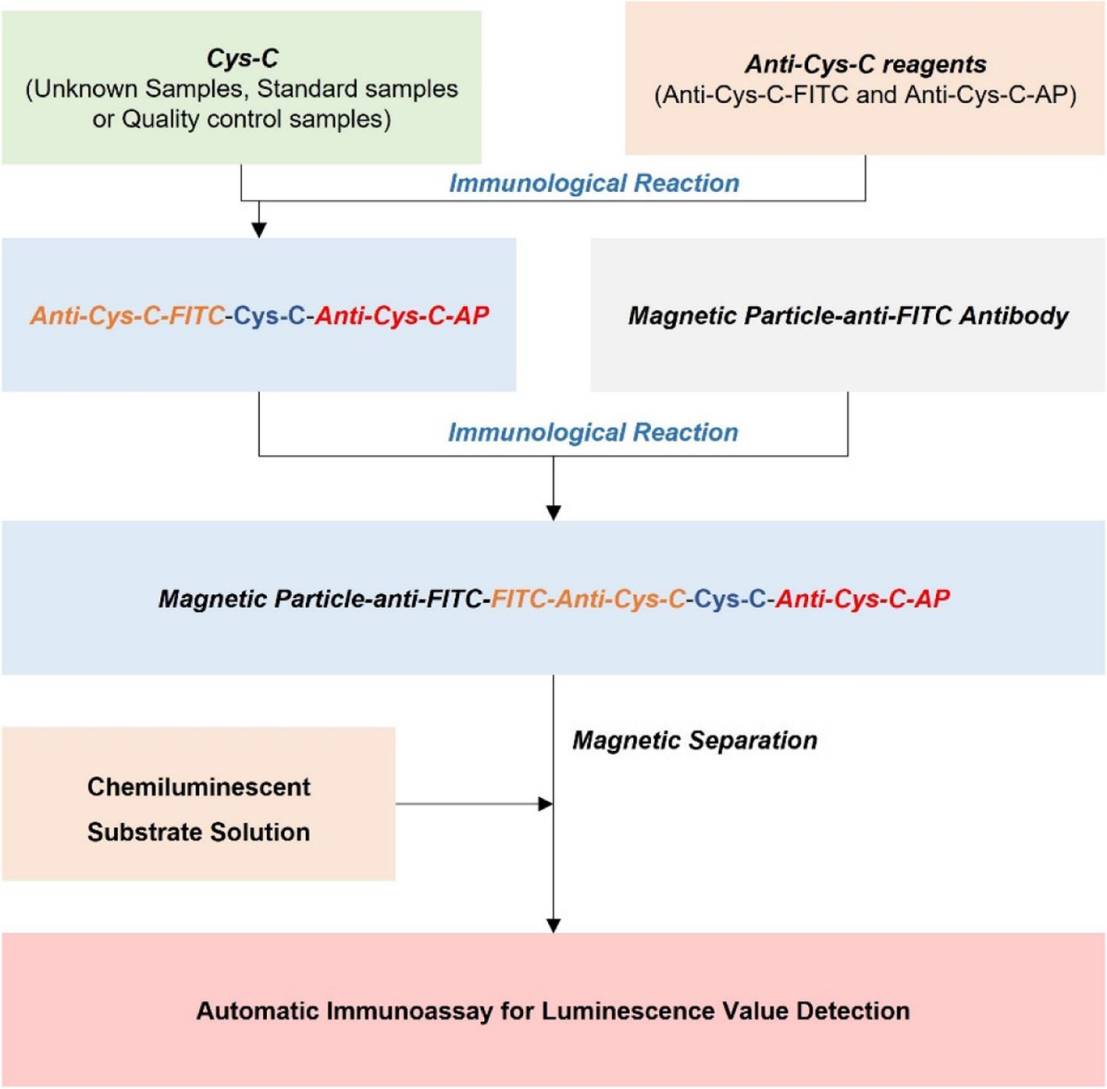
Scheme of the applied procedure

Samples were added into the reaction cup, then add 30 μL of anti-reagent A (anti-Cys-C-FITC) and 30 μL of anti-reagent B (anti-Cys-C-AP), mix well and react at 37℃ temperature bath for 5 min, add 30 μL of magnetic particle reagent, mix and react at 37℃ temperature bath for 5 min, add 300 μL of cleaning solution for chemiluminescence system, mix well, Repeat this step 3 times for magnetic separation and cleaning of the reactants. Finally, add chemiluminescent substrate solution for automatic immunoassay for luminescence value detection and concentration values are calculated if the calibrator curve is available (Fig. 6).

## Conclusion and discussion

According to the existing literature, Cys-C has been one of the sensitive indicators of early renal function injury in clinic [25, 27–29]. Currently, immunochromatography, chemiluminescence and colloidal gold methods are used to detect the content of Cys-C in clinical practice [24, 25]. Colloidal gold and immunochromatography cannot effectively meet clinical needs due to methodological limitations, poor sensitivity, accuracy and precision [30, 31]. Chemiluminescence method, immunochromatography method and colloidal gold method have obvious advantages in linear range, sensitivity, precision and accuracy, and good detection stability, detection with automatic instruments fast and convenient [31]. Chemiluminescence immunoassay of magnetic particles is a new analytical method which combines magnetic separation, chemiluminescence and immunoassay [31, 32]. This technology makes full use of the fast and easy automation of magnetic separation technology, the high sensitivity of chemiluminescence technology, and the specificity of immunoassay, showing an irreplaceable role in the field of biological analysis [32]. In this study, magnetic particle chemiluminescence technology was used to quantitatively detect the content of Cys-C in human serum, which has good performance, overall stability and systematic standard product tracing. The results showed that the method had good correlation with the Cys-C kit of Roche, which was widely used and recognized in the market. Through the study of the process and reaction system, we determined the source of raw materials and quality standards and inspection methods, developed the production technology, operating process, quality standards and detection methods of each component, established the product inspection standards and corresponding inspection methods of calibration kit, and evaluated the performance of the kit. In this study, in-house produced biomaterials such as magnetic particles, anti-Cys-C antibodies and anti-Cys-C antibody-AP couplings were critical to the performance of the reagent. Therefore, the process of antibody production and coupling needs to be further studied. Minimization of raw material batch differences helps maintain kit performance. This product is suitable for the determination of human serum samples, and its reliability for the determination of Cys-C concentration in other body fluid samples has not been fully confirmed. The test results of the kit are in good agreement with the disease and meet the clinical needs.

However, there are some limitations of this kit. This kit needs to be used with a special automatic magnetic particle chemiluminescence instrument, with high requirements for the instrument and high maintenance cost. In addition to this, this kit requires further validation in larger clinical samples if it is to be used in the marketplace.

## Data Availability

The data used to support the findings of this study are available from the corresponding author upon request.
